# Nuclear mechanobiology in confined cell migration

**DOI:** 10.1080/19491034.2026.2620879

**Published:** 2026-01-29

**Authors:** Hailee Patel, Simran Kaur, Richard B. Dickinson, Tanmay P. Lele

**Affiliations:** a Department of Biomedical Engineering, Texas A&M University, College Station, TX, USA; b Department of Chemical Engineering, University of Florida, Gainesville, FL, USA; c McFerrin Department of Chemical Engineering, Texas A&M University, College Station, TX, USA; d Department of Translational Medical Sciences, Texas A&M University, Houston, TX, USA; e Texas A&M School of Engineering Medicine, Texas A&M University, Houston, TX, USA

**Keywords:** Cellular mechanics, confined cell migration, mechanotransduction, nuclear deformation, nuclear mechanobiology, sub-nuclear structures

## Abstract

Nuclear deformation is a central challenge for migration of cells through confined spaces in the tissue interstitium. In this paper, we review studies on the mechanical roles of the nucleus in confined cell migration. We focus on mechanical force generation by the cytoskeleton on the nuclear surface, the properties of sub-nuclear structures in the process, and functional responses of the nucleus in response to mechanical forces, all in the context of confined cell migration. An emerging theme is that the nucleus acts not only as a barrier for confined migration, but also as a mechanoresponsive organelle whose deformation feeds back to modify cell behaviors. Deciphering these complex processes will be key to understanding how cells navigate complex tissues in development, immunity, and cancer.

## Introduction

Cell migration is crucial in development [1], tissue repair [2], and immune response [3]. Cell migration is also a necessary first step for the metastatic spread of cancer [4,5]. *In vivo*, mammalian cell migration typically occurs within crowded, confining interstitial tissue spaces as narrow as 1–10 µm [6]. Our understanding of how cells navigate these physical constraints remains limited because most studies of cell migration have focused on unconfined migration. Confined migration uniquely requires the nucleus to deform, as typical nuclear sizes (10–15 µm) exceed the size of narrow constrictions in tissue interstitium. Indeed, the deformation of the nucleus has been found to limit cell migration through constrictions [7,8]. As a result, studies have increasingly focused on the role of the nucleus in controlling cell migration in confinement. Deformation of the nucleus involves active mechanical force generation on the nuclear surface by the cytoskeleton (reviewed in [9,10]). These forces are balanced by mechanical stresses in intranuclear structures like chromatin and nuclear lamina. As such, there is considerable interest in literature in understanding the sources of mechanical force on the nuclear surface, the molecular transmitters of cytoskeletal force onto the nuclear surface, and the mechanical properties of nuclear components that resist these forces.

Furthermore, emerging studies have shown that nuclear deformation, by itself, may modulate nuclear transport, cause chromatin reorganization, and alter gene expression [11–15]. Deformation during migration can result in nuclear envelope rupture which can cause DNA damage, promote genome instability as well as promote invasive migration [16–18]. Thus, in addition to enabling cell migration through narrow constrictions, mechanical deformation of the nucleus may also feedback to regulate cellular processes and contribute to pathologies. Such adaptive processes are emerging as an exciting and under-recognized feature in nuclear mechanobiology.

Finally, the nucleus plays an important role in mammalian cell migration as a mechanical scaffold, even in non-confining contexts. The nucleus is typically positioned just rearward of the cell centroid of a motile cell such as a fibroblast [19–22]. The motile cell continually changes its shape as new lamellipodia form, and the trailing edge detaches. This results in a dynamic force balance on the nuclear surface [23]. Given its large size, the nucleus can act as an intracellular scaffold that transmits these forces across the cell [22,24]. Disrupting force transfer to the nuclear surface can disrupt cell migration even in unconfined environments. Furthermore, nuclear positioning by itself plays a definitive role in the process of development [21,25–27]. As such, the nucleus has diverse, context-specific, mechanical functions that deserve deeper study.

Here, we review studies that have focused on the mechanical roles of the nucleus in confined cell migration. We focus on mechanical force generation by the cytoskeleton on the nuclear surface, the properties of sub-nuclear structures in the process, and functional responses of the nucleus in response to mechanical forces, all in the context of confined cell migration.

## Lamin A/C limits migration through confinement

The nuclear lamina, a 15 nm thick meshwork of type-V intermediate filament lamin proteins that underlie the nuclear envelope [28,29], is a major determinant of nuclear resistance to mechanical deformation [8,30–38]. Unsurprisingly, cells with reduced levels of lamin A/C, a key structural component of the lamina, are able to pass through constrictions more easily than control cells [39]. For example, MDA-468 breast cancer cells with low levels of lamin A/C migrate faster compared to controls (transit times of 100 min for cells with reduced lamin levels versus 150 min for control) in 1 or 2 µm wide and 5 µm high constrictions [40]. The difference disappears in wider channels, highlighting that lamin A/C modulates migration specifically in confined spaces.

This effect appears to be general, because similar effects of lamin A/C depletion on confining cell migration have also been observed in mouse embryonic fibroblasts (MEFs) [8], glioblastoma-derived U251 cells, lung carcinoma-derived A549 cells and mesenchymal stem cells (MSCs) [41], HO-8910 ovarian cancer cells [42] as well as immune cells such as granulocytes and monocytes derived from human hematopoietic stem/progenitor cell cultures [43]. A summary of these and other studies, including the cell types, migration environments, constriction sizes, migration speeds, lamin A/C dependence, and nuclear envelope rupture incidence, is provided in Table 1. Likewise, exogenous expression of lamin A/C in cells lines with low levels of lamin A/C results in higher transit times through constrictions [41]. This has been shown for cancer cell lines (BT-549, MDA-MB-231 breast carcinoma cells, HO-8910 PM ovarian cancer, and HT1080 fibrosarcoma cells) [40,42,44] as well as neutrophil like HL-60 cells [45,46]. Also, phosphorylation of nuclear lamin A/C at serine 22 and serine 392 residues, which induces softening of the nucleus, promotes migration of MDA-MB-231 cells through pores in a Transwell migration assay [47].

**Table 1.** Studies on confined migration.

Cell Type	Migration Environment	Constriction Size (Pore size or Width x Height)	Transit Time or Migration Speed	Lamin A/C Dependence	NE Rupture	Reference
HT1080, MDA-MB-231, T-blasts, Human PMN	Collagen Matrices andTranswell	1–6 µm Pores 5, 3, 1, or 0.45 µm Pores	0-12 μm/min (Collagen Concentration dependent)	-	-	[7]
MEF, NIH 3T3	Microfluidic Device	2x5 µm^2^, 3x5 µm^2,^ 5x5 µm^2^ or 15×5 μm^2^	~170-240 min	Yes	-	[8]
MDA-MB-231, HT1080, MCF10A, U2OS	Microfluidic Device and Collagen Matrices	1x5 µm^2^, 2x5 µm^2^ or 15×5 μm^2^ 50-150 µm^2^, 5-20 µm^2^ or 1-15 µm^2^ Pores	-	-	Yes	[17]
RPE1, MCF10A, MDA-MB-231, MCF10DCIS.com	Microfluidic Device	2, 3, 4 µm high 10x50 µm^2^ regularly spaced gates connecting collagen-filled chambers (non-confining)	-	-	Yes	[18]
MDA-231, MDA-468, MCF7, SKBR3, HCC70, BT-549, BT-474, T47D	Microfluidic Device	1x5 µm^2^, 2x5 µm^2^ or 15×5 μm^2^	~100-200 min	Yes	-	[40]
A549, U251, MSC	Transwell	3 or 8 µm Pores	-	Yes	-	[41]
HO-8910, HO-8910PM	Transwell	3 or 8 µm Pores	-	Yes	-	[42]
Hematopoietic cells	Transwell	3, 5 or 8 µm Pores	-	Yes	-	[43]
A125, MDA-MB-231, HT1080, IFDUC1	Microfluidic Device and Collagen Matrices	1.7x3.7 µm^2^, 2.6x3.7 µm^2^, 2.8x3.7 µm^2^, 3.7x3.7 µm^2^, 4.4x3.7 µm^2^, 5.1x3.7 µm^2^, 5.8x3.7 µm^2^, 6.6x3.7 µm^2^, 7.3x3.7 µm^2^, 7.9x3.7 µm^2^, 8.4x3.7 µm^2^, 9.1x3.7 µm^2^, 9.9x3.7 µm^2^, 10.6x3.7 µm^2^ or 11.2x3.7 µm^2^ ~7 µm^2^ Pores	~0.1-2 μm/min (Constriction size dependent)	Yes	-	[44]
HL-60	Microfluidic Device and Transwell	5 µm wide channels 3 or 8 µm Pores	~10 ms	Yes	-	[45]
MDA-MB-231, HT1080	Collagen Matrices andTranswell	~2.9 μm Pores 3 µm Pores	~10-15 μm/hr	Yes	-	[47]
HT1080	Transwell	3 µm Pores	-	Yes	-	[50]
MFC, MSC	Transwell and Nanofibrous Scaffolds	3, 5 or 8 µm Pores ~150 μm thick	-	Yes	-	[52]
MDA-MB-231	Hydrogels with dumbbell microcavities	4x19 μm^2^, 7x19 μm^2^, 8x19 μm^2^, 10x19 μm^2^, 12x19 μm^2^, 15x19 μm^2^, 20x19 μm^2^ or 35x19 μm^2^	~25-40 μm/hr (Constriction size dependent)	-	-	[55]
MSCs, MDA-MB-231, L929	Microfluidic Device	3x10 μm^2^, 6x10 μm^2^, 10x10 μm^2^, 20x10 μm^2^ or 50x10 μm^2^	-	-	-	[56]
HT1080, HOS	Microfluidic Device	10x3 μm^2^ or 50x3 μm^2^	-	-	Yes	[57]
MDA-MB-231, hMSC	Microfluidic Device	3x10 μm^2^, 5x10 μm^2^, 10x10 μm^2^ or 20x10 μm^2^	-	-	-	[58]
HT1080, HEY, MEF	Microfluidic Device	2x7 µm^2^, 3x7 µm^2^ or 8x7 µm^2^	-	Yes	Yes	[59]
NIH 3T3, MEF, Glioblastoma cells, Skin fibroblasts	Microfluidic Device	2x5 µm^2^, 3x5 µm^2,^ 5x5 µm^2^ or 15×5 μm^2^	~125 min	Yes	-	[60]
MDA-MB-231, SUM159, MCF-10A	Microfluidic Device	3.5x10 µm^2^ or 10x10 μm^2^	~50 μm/hr	-	-	[61]
MDA-MB-231, MCF-7, MCF-10A	Collagen Matrices	7.3 ± 0.2 μm or 5.7 ± 0.2 μm Pores	-	-	-	[66]
BMSC	Transwell	8 µm Pores	-	-	-	[67]
HT1080, MDA-MB-231	Microfluidic Device and Collagen Matrices	1x5 µm^2^, 2x5 µm^2^ or 15×5 μm^2^ ~ 4-27 μm^2^ Pores	~100 min	-	Yes	[68]
Jurkat, PBL, MDA-MB-231	Transwell and Collagen Matrices	3, 5 or 8 µm Pores	-	-	-	[69]
HT1080, SW684, MDA-MB-231, A549, SK-MEL-28, HFF	Collagen and Cell Derived Matrices	-	~15-35 μm/hr	-	-	[78]
Human intestinal myofibroblasts and chondrocytes, HFF	Collagen and Cell Derived Matrices	-	~ 40 μm/hr	-	-	[79]
MEF, NIH 3T3	Microfluidic Device	2x5 µm^2^, 3x5 µm^2,^ 5x5 µm^2^ or 15×5 μm^2^	~150 min	-	-	[83]
MEF	Microfluidic Device	2x5 µm^2^, 3x5 µm^2,^ 5x5 µm^2^ or 15×5 μm^2^	-	-	-	[89]
MDA-MB-231, HT1080,	Collagen Matrix and Microfluidic Device	~ 2.3 μm or ~4.2 μm Interfibrillar distance 2.5 µm diameter	~ 0.3-0.5 μm/min (invasion) ~0.09 μm/min (constriction)	Yes	-	[90]
MDA-MB-231, HT1080, MEF	Microfluidic Device	1x5 µm^2^, 2x5 µm^2^ or 15×5 μm^2^	~25-50 min	-	-	[91]
U2OS, A549, MSCs	Transwell	3 or 8 µm Pores	-	-	Yes	[94]
NIH 3T3	Microfluidic Device	2x5 µm^2^, 3x5 µm^2,^ 5x5 µm^2^ or 15×5 μm^2^	-	-	Yes	[98]
Dendritic Cells, HL60-derived neutrophils	Microfluidic Channels	1x2 µm^2^, 1.5x3 µm^2^, 2x3.5 µm^2^, 3x4 µm^2^, 4x4 µm^2^, 5x4.5 µm^2^ or 7x4.5 µm^2^	~9-22 min (Constriction size dependent)	Yes	Yes	[99]
U2OS, HeLa	Micropillars	4.4 µm high pillars	-	-	Yes	[109]
Human Dermal Fibroblasts, MDA-MB-231, HT‐1080	Microfluidic Device and3D collagen‐based hydrogel matrices	10x10 µm^2^, 10x3 µm^2^ or 3x10 µm^2^ ≤1.5 µm^2^ Pores	-	-	Yes	[111]
A375, MDA‐MB‐231	Transwell	5 and 12 µm Pores	-	Yes	-	[113]
MDA-MB-231, BT-549, HT1080, RPE-1	Microfluidic Device and Collagen Matrices	1x5 µm^2^, 2x5 µm^2^ or 15×5 μm^2^ ~ 4-27 μm^2^ Pores	~70-120 minSpeed ~0.1 μm/min	-	Yes	[116]
U2OS, EC4, A549	Transwell	3 or 8 µm Pores	-	-	Yes	[117]
MDA-MB-231	Microfluidic Device	2x5 µm^2^ or 15×5 μm^2^	-	-	-	[118]
MDA-MB-231	Microfluidic Device	3x11 µm^2^, 5x11 µm^2^, 7x11 µm^2^ or 10×11 μm^2^	0.3-0.5 µm/s			[121]
MSC	Alginate Hydrogels	-	~2-80 μm/hr	Yes	-	[122]
HT1080, MDA-MB-231, A431, HOS, BRC-196	Microfluidic Device, Hydrogel-encapsulated microchannel array (HEMICA), Collagen and alginate gels	3×3 µm^2^, 10×3 µm^2^ or 10×10 µm^2^ 10×3 µm^2^ or 3×3 µm^2^	-	-	Yes	[126]
MDA-MB-231, SUM159, A431	Hydrogel-encapsulated microchannel array (HEMICA),	~4-60x10 μm^2^	~40-100 μm/hr (Constriction size and stiffness dependent)	-	-	[127]
MDA-MB-231, MDA-MB-231 BrM2, HOS, U87, HEK293, dermal fibroblasts, hAOSMC, SUM159	Microfluidic Device	3.5x10 µm^2^ or 10x10 μm^2^	~50-100 μm/hr (Extracellular fluid viscosity dependent)	-	-	[128]
MDA-MB-231, SW1990, Pa0C3, HOS	Microfluidic Device	3x10 µm^2^, 6x10 µm^2^, 10x10 µm^2^, 20x10 µm^2^, or 50x10 µm^2^	~ 35 µm/hr	-	-	[129]
U2OS, C2C12, A549	Transwell	2-8 µm Pores	-	-	Yes	[130]
Dendritic cells, T cells, Human peripheral blood polymorphonuclear leukocytes (PMNs), 3T3 Swiss fibroblasts	Microfluidic Device and Collagen Matrices	2x4 μm^2^, 3x4 μm^2^, 4x4 μm^2^ or 5x4 μm^2^ 1-5 μm Pores	-	Yes	-	[131]
S180, MDA-MB-231, CH2879	Microchannel Device	3x10 μm^2^, 3x6 μm^2^ or 50x10 μm^2^	~16 μm/hr	-	-	[132]
HOS, MCF7, MDA-MB-231, MCF10A	Microfluidic Device	3x10 μm^2^, 6x10 μm^2^, 10x10 μm^2^, 20x10 μm^2^ or 50x10 μm^2^	~30-74 μm/hr	-	-	[133]
C2C12, Rh30, hMSCs, Human primary skeletal muscle stem cells	Transwell	3 or 8 µm Pores	-	-	Yes	[134]
A549, HT1080, BT-549	Microfluidic Device	1x5 µm^2^, 2x5 µm^2^ or 15×5 μm^2^	~60 min	-	Yes	[135]
U2OS, U251, A549	Transwell	3 or 8 µm Pores	-	-	Yes	[136]

Consistent with the idea that the mechanical properties of lamins are responsible for these effects, expression of progerin – a shortened, permanently farnesylated laminA variant missing 50 amino acids near it’s C-terminus due to aberrant splicing of LMNA transcript and linked to Hutchinson-Gilford progeria syndrome – stiffens the nucleus [48] and prevents migration through micro-posts spaced 6 µm apart, despite exhibiting normal migration in unconfined environments [49]. Some studies suggest that the ratio of lamin A to lamin B levels is important in determining the migratory response of cells in confinement. A549 cells and MSCs, in which this ratio is more than 1, migrate through 3 µm pore Transwell filters to a lesser extent than U251 cells, in which the ratio is less than 1 [41].

## The taut nuclear lamina mechanically impedes confined cell migration

As lamin A/C depletion causes a softening of the entire nucleus [31,37], the energetic cost of mechanically deforming the whole nucleus to fit into constrictions will be lower in lamin A/C depleted cells compared to controls. Cells with stiffer, lamin A/C containing nuclei are predicted to be impeded in migration through confinement, while cells with softer nuclei are predicted to pass through more easily. Thus, the nuclear lamina is thought to *mechanically* impede cell migration through confinement by limiting nuclear deformation [50–52], rather than through influences on the cell migration machinery itself.

Mechanical deformation of a spherical nucleus at constant volume would require an increase in the nuclear surface area because the sphere has the smallest surface area for a given volume. Consequently, compressing or elongating the sphere can only occur if the nuclear lamina stretches to accommodate the additional surface area. However, the lamina is likely too stiff to be significantly stretched by cellular forces [53,54]. How then are cells able to deform the nucleus to fit into the constriction? One possibility is that nuclei shrink in volume as they migrate into confinement, as reported in MDA-MB-231 breast cancer cells [55] and L929 fibroblasts [56], but others have observed an increase in nuclear volume in confinement in HT1080 fibrosarcoma cells [57] and MDA-MB-231 cells [58]. Nuclei in HT1080 cells and HEY ovarian cancer cells maintain constant volume as they enter and exit a constriction [59], and others also have reported that nuclear volume remains constant during mouse embryonic fibroblast migration in constrictions [60]. The nucleus has been reported to change its volume typically in confinement channels that are significantly longer than nuclear and cell dimensions, which may induce adaptive effects as the nucleus is forced into confinement for long periods of time. For example, sustained migration of MDA-MB-231 breast cancer cells in confining channels of 200 µm length was accompanied by an increase in nuclear volume [61]. In contrast, confinement lengths of the order of the nuclear diameter typically allow the nucleus to exit and enter in relatively short times from 30 min to 1 hr, which may not be long enough to induce secondary adaptive effects on nuclear volume and/or surface area. Both short confinement and long confinement likely model distinct aspects of physiologically relevant confinement. Short confinement lengths are typical for cells that migrate through discrete pores of the extracellular matrix or when extravasating from the vasculature. Long confinement may be more typical for cells that migrate over extended distances such as during collective cell migration along aligned collagen fibers, neural crest migration, or leukocyte movement, where the cellular environment imposes sustained physical boundaries.

**Figure 1.**
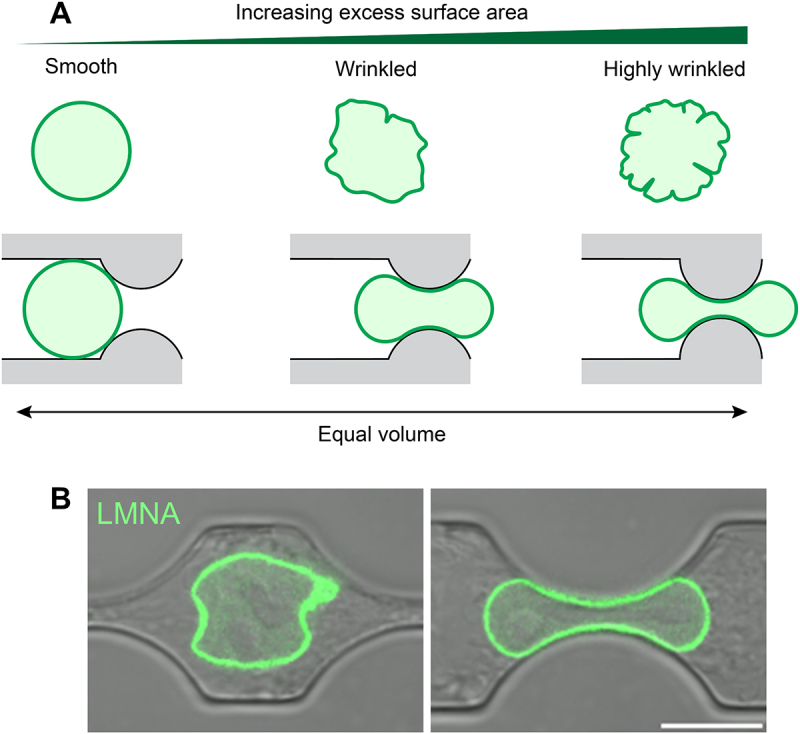
Role of laminar excess area in excluding cells from constrictions. (A) (left) a sphere will not deform into the constriction under the constraint of constant surface area and volume and will be excluded from it. (middle) excess area, visible as wrinkles in the rounded shape, will allow the nucleus to deform at constant surface area and volume and fit into the constriction. (right) large amount of excess area in the nucleus will allow the nucleus to deform and fit into narrower constrictions. (B) images of an HT-1080 cell expressing GFP lamin A, overlaid on DIC images of the cell and constriction channel, as it migrates into a 3 μm ×7 μm constriction (right). The same nucleus is shown on the left before it enters the constriction. Modified from Mckee et al., Science advances, DOI: http://doi.org/10.1126/sciadv.Ads6573, 2025, with permission from AAAS.

For a nucleus that obeys constraints of constant surface area and volume during deformation [59], only if sufficient excess laminar surface area is present in the rounded shape of the nucleus – defined as the laminar area more than that of a sphere with the same volume – will the nucleus deform to fit through narrow constrictions without changing its volume or surface area (Figure 1A). The excess area can be observed as wrinkles in the rounded shape of the nucleus (Figure 1B) and develops when the nuclear envelope and lamina assemble around non-spherically packed chromosomes post-mitosis [62]. Narrowing of the shape of a wrinkled nucleus at constant volume will cause an unwrinkling of the lamina (Figure 1B). The greater the excess area, the more the lamina can be unwrinkled, enabling more extensive nuclear deformation and passage through narrower constrictions. Consistent with the above predictions, nuclei successfully entering constrictions exhibit a higher excess laminar surface area compared to excess area in the unconfined population. This suggests that nuclei with insufficient excess area are altogether excluded from the constrictions.

Depletion of lamin A/C is predicted to remove the areal constraint altogether, as the lamina can stretch and even tear in the absence of lamin A/C [63]. Consistent with this, lamin A/C depletion shifts the excess area distribution to higher values in cells in constrictions [59]. Thus, excess area may determine whether a given nucleus will fit into a constriction or not (provided the nucleus deforms at constant area and volume). This explains *how* the nuclear lamina might mechanically limit migration through confinement – through a process of exclusion of low excess-area containing cells.

**Figure 2.**
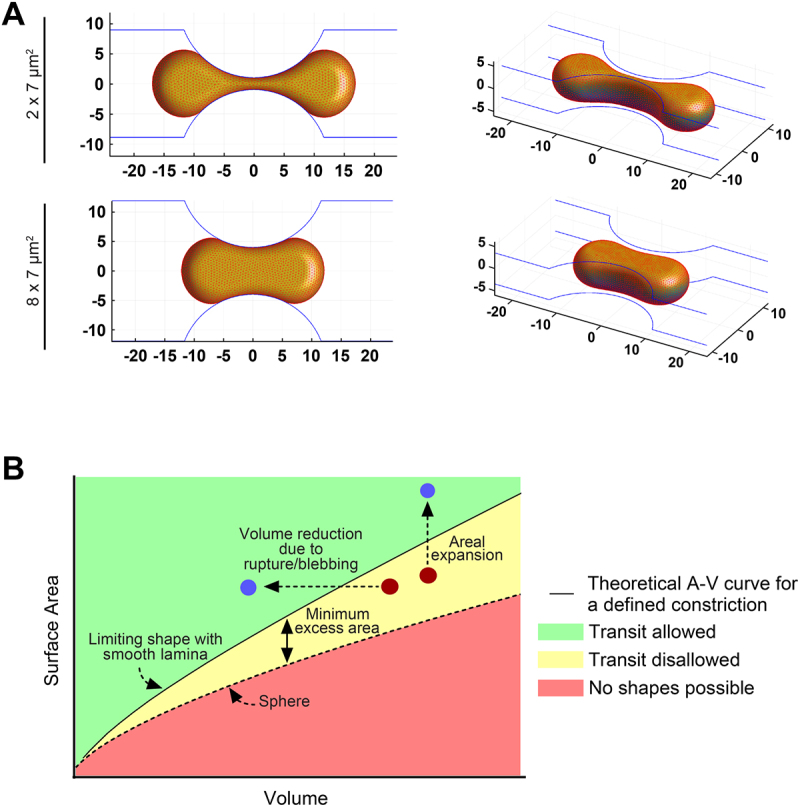
Explaining the varying outcomes for a nucleus in confined migration. (A) computational predictions of nuclear shapes with minimum surface area for a given volume in constrictions of defined size. (B) schematic depicts different outcomes (volume loss due to rupture or blebbing, and areal stretching) relative to the limiting area–volume curve (solid line) calculated using the computational model. Modified from Mckee et al., Science advances, DOI: http://doi.org/10.1126/sciadv.Ads6573, 2025, with permission from AAAS.

Assuming the excess area mechanism described above is valid, a geometric computational model [64] can be used to calculate the nuclear shape with minimal surface area for a given volume that fits into the constriction [59] (Figure 2A). The predicted shapes recapitulate the observed hourglass shape of nuclei in constrictions (compare with hourglass nuclear shape in Figure 1B). Furthermore, the model allows the calculation of a limiting area–volume curve (solid black curve in Figure 2B). Nuclei that are above this curve will pass through constrictions (green region in Figure 2B), while those below it will be excluded from the constrictions (yellow region) unless they lose volume or expand laminar area. This curve can then be used to rationalize the different outcomes that have been reported for nuclei in constrictions, as indicated in Figure 2B. These outcomes include rupture which reduces the nuclear volume at constant area, stretching of the surface area (which can occur in the absence of lamin A/C), and blebbing, which reduces the effective volume of the nucleus. All these outcomes allow a nucleus with insufficient excess area to transit the constriction.

## Chromatin modifications alter migration through confinement

Given that the nucleus appears to mechanically limit confined migration, it is not surprising that chromatin, which contributes to the resistance to deformation of the nucleus [32,65], may also play a role. However, opposing effects of modifying chromatin on confined migration have been reported. Treatment of adult meniscal fibrochrondrocytes (MFCs) with Trichostatin A (TSA), a histone deacetylase inhibitor which decondenses chromatin, promoted cellular infiltration into dense fibrous tissues [52]. Similarly, chromatin decondensation in MDA-MB-231 or MCF-7 breast cancer cells resulted in increased invasion depth in dense 3D collagen matrices of ~5.7 micron pore size [66]. In contrast to these studies, chromatin decondensation by TSA treatment reduced bone-marrow derived mesenchymal stem cell (BMSC) migration through 8 µm Transwell pores [67]. Likewise, treatment of HT1080 fibrosarcoma cells with 3-Deazaneplanocin A (DZNep), a histone methyltransferase inhibitor which reduces heterochromatin levels, decreased migration speed through both 15 × 5 µm^2^ control and 2 × 5 µm^2^ sized constrictions. Notably, the reduction in speed was less pronounced in control channels [68]. Similarly, silencing H3K4 methyltransferase components WDR5 or RbBP5, significantly reduced migration of lymphocytes through both 3D collagen matrix and 3 µm pore Transwells [69]. Also, decoupling heterochromatin from the nuclear periphery inhibited constrained migration *in vivo* in *Caenorhabditis elegans* larval development [70]. Consistent with these studies, increased H3K9me2/3 levels promoted chromatin compaction and enhanced motility in T-cells [71]. The reasons for the disagreement on the role of chromatin in confined migration are presently unclear. While nuclear softening caused by chromatin decondensation has been proposed to promote confined migration in some contexts [52], it is possible that chromatin modifications give rise to secondary effects (such as altered migratory signaling pathways) in others.

## Mechanical forces that move the nucleus through confined spaces vary with the mode of cell migration

Cells must actively position the nucleus, their largest organelle, in order to migrate effectively. They have evolved diverse mechanisms to position the nucleus (reviewed by us in [9]), with qualitatively different force generation modes: shear forces generated by kinesin or dynein microtubule motors [25,72–74] or by retrogradely flowing actomyosin flow [75,76], tensile forces through actomyosin contraction [22,23,77], and forces due to a front-to-back pressure gradient [78,79]. These distinct force mechanisms can be harnessed by the cell depending on the context and on whether the migratory mode is amoeboid or mesenchymal.

**Figure 3.**
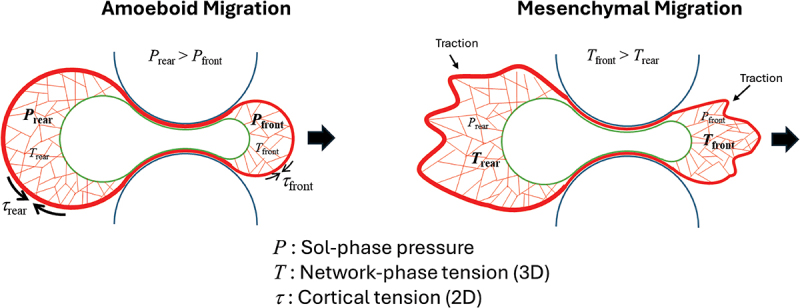
Model for translocation of the nucleus through a constriction during confined migration. Nuclear translocation is driven by a back-to-front difference in cytoplasmic stresses on the nuclear surface. The net cytoplasmic stress is the difference of pressure in the sol-phase (P) and the tension in the network phase (T), the latter of which is transmitted to the nuclear envelope via nesprins. Sol-phase pressure is generated by contraction of the actomyosin cortex (i.e. The Laplace pressure at the plasma membrane). In amoeboid migration, a front-to-back difference in cortical (2D) tension (τ) creates the pressure gradient that drives cytoplasmic flow and nuclear translocation. In mesenchymal migration, nuclear translocation results from a front-to-back difference in traction forces transmitted from the surrounding extracellular matrix (or substratum) to the nuclear surface through the actomyosin network. A front-to-back difference in stress transmission to the nuclear surface could arise from a difference in traction forces, in actomyosin activity or in the surface density of nesprin linkages.

Translocation of the nucleus through a constriction requires a force difference between the front and back of the nucleus. Accounting for the biphasic nature of the cytoplasm, which consists of a viscoelastic interconnected actomyosin network (gel phase) embedded in a cytosolic fluid (sol phase), the net local stress on the nuclear surface is the difference between the tensile stress from the contractile actomyosin network phase (linked to the nuclear surface through nesprins) and the pressure of the sol phase (the cytosolic fluid component) (Figure 3) [80,81]. Pressurization of the sol phase is achieved by Laplace pressure generated by actomyosin cortical tension. Amoeboid migration is commonly attributed to greater cortical tension (and thus higher pressure) at the cell rear, which drives forward cytoplasmic flow and nuclear translocation. In contrast, mesenchymal migration involves traction forces that are transmitted through the nucleus from front to back via the actomyosin network permeating the cytoplasmic volume [22,82]. The force difference driving nuclear translocation in confined mesenchymal migration could therefore arise from greater actomyosin activity at the cell front, where polymerization is more active [23], and/or greater stress transmission at the front due to a higher density of cytoskeletal linkages at the nuclear surface [83]. In principle, nuclear translocation in confinement could be driven by both a sol-phase pressure difference and a network-phase tension difference, with one or the other mechanism predominating during amoeboid- or mesenchymal-type migration. Moreover, cells migrating through confined gaps remodel the actin cortex and retain a memory of prior confinement events [84]. The mechanical state of the cortex at one confinement influences cell morphology and migratory capacity at subsequent confinements, demonstrating a direct link between past confinement and future adaptive potential.

Early studies of mesenchymal migration in unconfined motile fibroblasts showed that nuclear positioning is accomplished by a tug-of-war between actomyosin pulling forces generated in between the nuclear surface and the leading edge, and those generated in between the nuclear surface and the trailing edge [22,23]. In this picture, an increase in actomyosin force close to the leading edge where new F-actin filaments are formed causes an imbalance between the pulling forces on the nucleus from the trailing edge and the forces from the leading edge, resulting in motion of the nucleus toward the leading edge. The transmission of these pulling forces to the nuclear surface requires a linkage between the F-actin cytoskeleton and the nuclear surface mediated by the Linker of Nucleoskeleton and Cytoskeleton (LINC) complex [22,23,85], which spans the nuclear envelope and consists of inner nuclear membrane Sad1 and UNC-84 (SUN) domain containing proteins, and the outer nuclear membrane Klarsicht, ANC-1, and Syne homology (KASH) domain-containing nesprin proteins [77,86–88]. Among these, the giant isoforms of nesprin-1 and nesprin-2 directly bind F-actin.

Interestingly, actomyosin pulling forces between the leading edge and the nuclear surface have been similarly implicated in the migration of mouse embryonic fibroblasts (MEFs) in 2 µm, 3 µm, or 5 µm sized constrictions [83,89]. The F-actin binding nesprin-2 KASH protein was observed to redistribute to the leading edge of the confined nucleus, and this redistribution was required for nuclear migration into the constriction. Thus, F-actin based pulling forces on the nuclear surface from the front of the cell likely move the nucleus forward and into the constriction in MEFs. Others have suggested that dynein motors anchored to the nuclear surface by the LINC complex, pull the nucleus along microtubules radiating from the centrosome into the constriction [90]. However, pressure from the rear of cells due to contraction of the rearward actomyosin cortex, which creates a pressure difference between the front and back of the cell, may also contribute to the force balance in narrower constrictions [55]. Cancer cells like MDA-MB-231 cells, HT1080 cells, or HOS cells, which have the capacity for amoeboid migration, may move the nucleus into constrictions through such a pressure difference, while MEFs, which migrate exclusively mesenchymally, may use a purely tensile mechanism [57,91]. A similar mechanism, based on front-to-back pressure gradients, has been proposed for moving the nucleus through 3D matrices in which the nucleus is proposed to act as a piston [78,79]. Studies with *C. elegans* suggest that while dynein may pull the nucleus through constricted spaces along microtubules toward their minus ends in a LINC-complex dependent manner, actomyosin contraction from the rear may push the nucleus forward in a LINC-complex independent manner [88]. Also, cells lacking Cytoplasmic Linker-Associated Proteins (CLASPs) were unable to migrate directionally in microchannels of 2–2.5x4 µm constrictions, and nuclei underwent frequent rupture events [92]. Consistent with this, CLASP2 depletion in RPE1 cells impaired migration through 4 µm confinement channels [93].

## Biological effects of nuclear deformation in confinement

### Nuclear envelope rupture

The large deformations of the nucleus as a cell migrates through confinement can rupture the nuclear envelope [16,17], mixing cytoplasmic and nuclear contents, and causing deleterious effects such as DNA damage [94–96]. Nuclear envelope ruptures have been observed in diverse cell types including cancer cells [16,17,97], fibroblasts [98], and dendritic cells [16,99], and they occur even in unconfined cells when they lack nuclear lamins [100,101].

A single lipid membrane can undergo an areal stretch of only around 2–5% before it will rupture, corresponding to a rupture tension of around 8–10 mN/m [102]. The resulting hole size in single membrane tends to be of the order of a few nm [103,104]. Yet, nuclear envelope hole sizes post-rupture in cells have been reported to be of the order of 100 nm [17,105]. This may be due to the unique mechanical properties of the double-membrane fused structure of the envelope [106–108], which could give rise to donut-shaped holes that are stable at much larger sizes than holes in single membranes [105].

Envelope rupture frequency is higher at smaller confinement widths [17]. Ruptures typically occur at the leading edge of the nucleus and are preceded by the formation of membrane blebs that separate from the nuclear lamina and eventually burst. Bleb formation may be promoted by chromatin herniation that exerts an outward pressure on the nuclear envelope [109,110]. Loss of lamin A/C promotes the tendency for rupture [17], while vimentin intermediate filaments protect nuclei from rupture [96].

Upon rupture, DNA damage is induced by exposure of DNA to endoplasmic reticulum (ER)-associated exonuclease TREX1 after envelope integrity is breached [18]. This DNA damage has been shown to promote invasive migration in human breast cancer cells. Frequent rupture in fibrosarcoma cells can also induce p53 activation and cell apoptosis, but cells can eventually adapt by excluding Yes-associated protein (YAP) – a mechanosensitive transcriptional co-activator and major effector of the Hippo signaling pathway that regulates genes involved in cell proliferation, survival, and migration – from the nucleus, reducing the incidence of ruptures and suppressing p53-mediated cell death [111].

Ruptures of the nuclear envelope are repaired by endosomal sorting complexes required for transport-III (ESCRT-III) machinery [16,17]. The recruitment of repair protein machinery to ruptures is preceded by rapid recruitment of proteins like barrier-to-autointegration factor (BAF) and Lamin C to the site of rupture [98,112].

## Chromatin remodeling

Nuclear deformation that accompanies confined cell migration can lead to chromatin rearrangement and function that can last from hours to days [39,68]. Migration of HT1080 fibrosarcoma cells, skin fibroblast or MDA-MB-231 breast adenocarcinoma cells through three rows of 2 × 5 µm^2^ sized repeating constrictions in a microfabricated device resulted in increased heterochromatin (H3K9me3, H3K27me3) and reduced chromatin accessibility, which in turn decreased global transcription [68]. This remodeling promoted cell migration in confined 3D environments and impairing these changes inhibited migration. In contrast, heterochromatin levels reduced in cell migrating through high-concentration 3D collagen gels [68,69].

Notably, the effects of confined migration on chromatin are not necessarily transient. Repeated migration through narrow constrictions can have long-lasting effects on chromatin organization. For example, A375 melanoma and MDA-MB-231 cells, which went through multiple rounds of confined migration, exhibited stable changes to chromosomal spatial compartmentalization as measured in Hi-C experiments [113]. These changes further correlated with stable changes to gene expression, including in metastasis-associated pathways (TGF-β, EGFR) and adhesion-related genes such as CADM3 and ITGA9. CCRF-CEM and Jurkat cells that underwent three sequential rounds of confined migration exhibited irregular nuclear morphology with irregular lamin B1 distribution [114]. These cells showed altered expression of genes involved in cell-cycle regulation, DNA-damage response, protein modification pathways. These cells also exhibited increased resistance to apoptosis, increased penetration into 3D collagen matrices and, surprisingly, decreased invasiveness in vivo. Cell migration through confinement can also alter chromatin conformation. For example, neutrophil migration through constrictions can cause a depletion of short-range chromosomal contacts in compartments enriched for heterochromatin [115]; such remodeling was not observed in transcriptionally active compartments. Thus, heterochromatin remodeling, which occurs at the periphery of the nucleus, may have a protective role that dampens remodeling in euchromatin deeper in the nucleus.

## Nuclear condensate and phase separation

Nuclear deformation in confined migration can induce DNA damage independent of rupture by causing stalling of the replication fork [116,117]. Also, mechanical deformation of the nucleus during migration can modulate chromatin-embedded nuclear condensates [118,119]. For instance, deformation of the nucleus through a 2 μm × 5-μm-wide confinement deformed and coalesced nuclear condensates such as nucleoli and nuclear speckles in human MDA-MB-231 breast cancer cells. Notably, the advancing edge of the nucleus displayed a more homogenous chromatin distribution, whereas the trailing edge was more heterogeneous, with pockets of lower chromatin density. Condensates preferentially nucleated and grew in size in the trailing half of the migrating nucleus (compared to the advancing half) [118]. This mechanical asymmetry mirrors prior observations that constrictions enhance chromatin compaction while excluding mobile nuclear factors, such as DNA repair proteins, from regions of high density [117]. In contrast, heterochromatin protein alpha (HP1α) condensates shift significantly toward the nuclear center in response to rapid vertical confinement of 2–8 µm for ~15 min in fetal lung fibroblasts (IMR90) but not in human cervical cancer (HeLa) cells, indicating a cell-type dependent response [120]. Interestingly, the HP1α condensates were observed to decrease both in size and number for both cell types under confinement, likely due to force-dependent disintegration of the condensates. Similarly, moderate confinement of MDA-MB-231 through microchannels 11 µm ×7 wide or 11 µm ×10 µm wide resulted in twofold increase in nuclear paraspeckles, which decrease to baseline levels when cells exit the confined region, with no change in paraspeckle expression during extreme confinement (3 or 5-µm-wide channels) [121]. Paraspeckle formation was also nearly doubled with near tripling in total paraspeckle area with the 11 µm × 10-µm-wide confinement. These paraspeckles were observed to be localized at the leading edge of the migrating cells, localizing in euchromatin enriched regions.

## Signaling pathways

The significant nuclear deformations in confined migration can result in interesting effects that impact cell migratory pathways in unusual ways. As mentioned above, upon confinement, the nucleus can act as a piston to pressure the front of the cell. Such pressure gradients can open ion channels in long protrusions at the leading edge of the cell in confining pores [122], resulting in stabilization of these protrusions. Thus, the nucleus may help power migration through confinement. Likewise, compression of nuclei as they move into constrictions may feedback to modify the very forces that deform and position the nucleus. This possibility is supported by experiments in which stationary cells were compressed vertically in a confinement device [123]. Compression unfolded the nuclear envelope, triggering a signaling cascade with the release of calcium ions and activation of the cytosolic phospholipase A_2_ enzyme (cPLA_2_), that promoted actomyosin contraction. In line with this, Arp2/3 mediated nuclear envelope unfolding and tensioning in response to confinement activated cPLA_2_, driving upregulation of chemokine receptor CCR7 which induced shape sensing in dendritic cells [124]. Similarly, nuclear envelope or inner membrane stretching during confinement activates the same cPLA_2_-calcium-arachidonic acid signaling cascade, promoting cortical myosin II recruitment and cell contraction [125]. This mechanism enables cells to generate contractile forces required for nuclear translocation through constrictions and to adjust motility in response to confinement. Along similar lines, confined migration recruits Ect2, cofactor of the scaffolding protein anillin, to the plasma membrane where it mediates the activation of myosin-mediated contraction that feeds back to further drive migration; nuclear rupture further amplifies this signaling response [126]. Also, nuclear deformation-induced unwrinkling of the nuclear lamina has been proposed to modulate nuclear surface tension and promote YAP localization to the nucleus [53]. Whether such changes to the physical state of the nucleus also modify mechanical force generation in confined migration remains an open question.

## Summary

Confined cell migration represents a distinct mechanical mode of cell migration which involves deformation of the nucleus to fit into narrow constrictions. This area of research is rapidly expanding, and brings together microfluidic technologies, 3D ECM models of confined migration, biophysical modeling and molecular cell biological investigations. Significant progress has been already achieved in understanding the mechanics of nuclear deformation, the mechanisms of force generation on the nucleus, and unique deformation-induced biological effects such as envelope rupture, modification of chromatin conformation and alteration of signaling pathways. This mounting evidence suggests that nuclear deformation in confined migration can induce functionally important effects such as DNA-damage, invasive cancer cell migration, and alteration of signaling pathways. The accumulating evidence already raises the prospect of therapeutic targeting of deformation-induced pathways for arresting or reducing the invasive migration of cancer cells.

At the same time, important conceptual gaps remain. In particular, much of the field continues to treat the nucleus primarily as a viscoelastic material, without accounting for the physiologically relevant, slow time scales of nuclear deformation during confined migration over which the nucleus behaves more like a drop. On these time scales, nuclear mechanics appear to be governed by a threshold behavior of the nuclear lamina: effectively zero surface tension when the lamina is wrinkled, and a finite surface tension when the lamina is smooth. An important, unexplored implication is that nuclear functions such as nucleocytoplasmic transport and regulated gene expression may be sensitive to laminar surface tension itself, rather than to nuclear deformation or strain alone. Clarifying these relationships will require approaches that explicitly connect nuclear geometry, laminar state, and functional nuclear responses during confined migration. In parallel, establishing more realistic in vitro models that reflect tissue-specific patterns of confinement, including pore sizes and 3D geometries, is required for fully understanding nuclear mechanics in confinement.

## Data Availability

This article is a review of previously published literature. No new data was generated or analyzed in support of this article. All data has been referenced and available at the cited source.
